# Nanobody-based characterization of Galectin-13 reveals immunomodulatory and tumor-promoting functions

**DOI:** 10.1016/j.jbc.2026.113467

**Published:** 2026-08-19

**Authors:** Camille Fuselier, Philippine Granger Joly de Boissel, Rita Nehmé, Alex Paré, Fanny Fronton, Sammy Ajarrag, Duong T. Bui, Marlène Fortier, Alyssa Dumoulin, John S. Klassen, Nicolas Doucet, David Chatenet, Yves St-Pierre

**Affiliations:** 1 INRS-Centre Armand-Frappier-Santé Biotechnologie, Laval, Quebec, Canada; 2 Department of Chemistry, University of Alberta, Edmonton, Alberta, Canada

**Keywords:** galectins, nanobodies, placental galectin, chemokine, hepatic carcinoma, apoptosis

## Abstract

Galectin-13 (GAL-13), also known as placental protein 13, is a lectin primarily studied in the context of pregnancy and immune regulation at the maternal-fetal interface. However, its potential roles in other tissues, including cancer, remain largely unknown. In this work, we provide new insights into the role of this protein while developing a comprehensive set of research tools to facilitate future studies. Our transcriptomic analyses reveal that GAL-13 reprograms gene expression in HepG2 liver cells, suggesting functions beyond placental physiology, potentially linked to inflammatory and tumor-related signaling. We generated a panel of 10 high-affinity nanobodies that enable detection, neutralization, and affinity-based purification of GAL-13, overcoming long-standing challenges in obtaining a soluble, functional form of the protein. Importantly, this large nanobody panel also provides an opportunity to map the functional epitopes of GAL-13, including those outside its classical carbohydrate recognition domain (CRD). The purified GAL-13 retains its biological activities, notably inducing apoptosis in Jurkat T cells and stimulating *CCL20* expression in HepG2 cells. This *de novo* induction of *CCL20* was inhibited by our nanobodies, demonstrating their utility in dissecting GAL-13-mediated effects. Moreover, we observed extensive cross-reactivity of several nanobodies with GAL-16, reflecting the strong structural similarity between these two placental galectins and underscoring the need for careful validation of reagent specificity. Altogether, by integrating methodological innovation with functional analyses, our study provides essential molecular tools to advance GAL-13 research and reveals an emerging role for this understudied lectin in cancer biology.

Galectins (GALs), a family of β-galactoside-binding proteins, have attracted significant attention due to their roles in critical cellular processes driving cancer progression (1). They are composed of a carbohydrate recognition domain (CRD), which plays a crucial role in their ability to bind specifically to glycosylated structures on cell surfaces and regulate underlying signaling pathways (2, 3, 4). Thus, galectins exert diverse effects on tumor progression, particularly by inducing the expression of genes involved in angiogenesis, cell adhesion, migration, and proliferation (5, 6, 7). Moreover, certain galectins, such as GAL−1, −3, −7, and −9, are known to induce local and systemic immune suppression through various mechanisms, including modulation of immune cell function and induction of T cell apoptosis. These functions have attracted considerable interest in developing new cancer treatments, especially for types with limited treatment options.

Galectins have shown promise as diagnostic and prognostic markers in cancer. Their expression levels in tumor tissues or body fluids, such as blood or urine, have been correlated with specific cancer types, tumor stage, and patient outcomes (8, 9, 10). However, research has focused predominantly on a subset of galectins, primarily GAL−1, −3, −7, and −9, leaving a knowledge gap regarding the functions of other galectins. GAL-13, also known as Placental Protein 13 (PP13), is a prototypic, relatively newly studied galectin with crucial roles in placental development (11, 12, 13, 14). Unlike most galectins, GAL-13 does not bind the typical sugar structures recognized by this protein family but has demonstrated binding activity to mannose, N-acetyl-lactosamine, and N-acetyl-glucosamine. Its lack of inhibition by various sugars common to most galectins sets it apart (15, 16).

Emerging evidence suggests that placental galectins may also be present in other tissues, including cancerous tissues (11, 17, 18). In the case of GAL-13, for instance, it is highly expressed in the spleen, bladder, and even in benign and malignant tumors from various organs, including the liver (11). However, our understanding of the pro-tumorigenic functions of GAL-13 is limited. Recent studies have implicated GAL-13 in promoting the survival of regulatory neutrophils, increasing PD-L1 expression, and stimulating the release of matrix metalloproteinase-9 (MMP-9), an enzyme known to play a central role in tumor progression (19). Other studies have shown that GAL-13 can induce T cell apoptosis and promote CXCL8 (IL-8) production, a key mediator of angiogenesis and neutrophil recruitment (20). These findings suggest that GAL-13 may be involved in the immune response within the tumor microenvironment, providing a potential link between placental biology and cancer physiology (21). However, the absence of appropriate research tools has greatly impeded studies on this galectin's role in cancer and other diseases. In this work, we addressed these challenges and provide new insights into the role of this protein in modulating the expression of genes associated with cancer progression, and developed a comprehensive set of research tools to enable future studies.

## Results

### GAL-13 induces transcriptional reprogramming in HepG2 liver cancer cells

**Figure 1:**
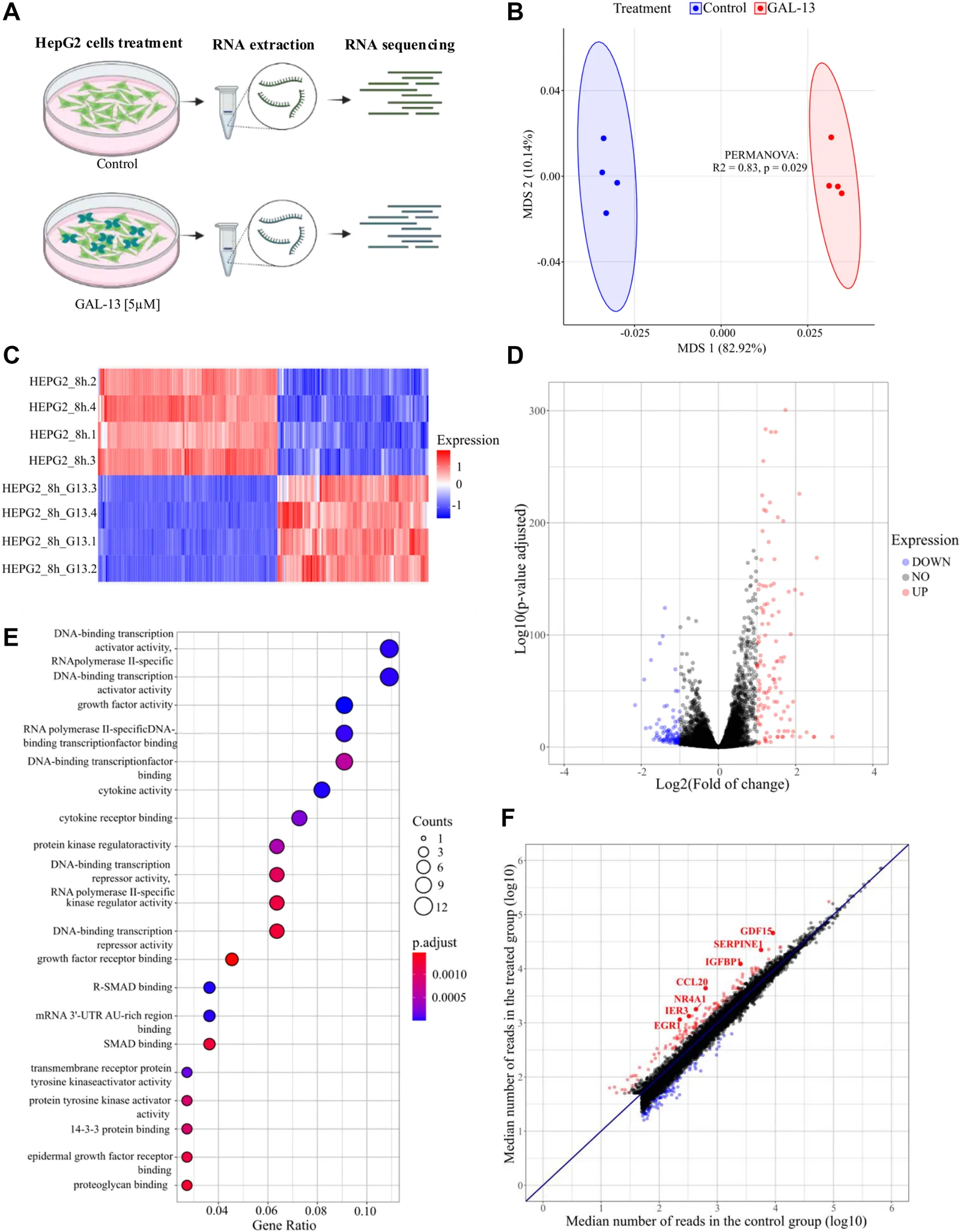
**GAL-13 induces gene expression associated with cancer.***A*, overview of the bulk RNA-Seq experiment. *B*, principal component analysis of the RNA-Seq data shows clear separation between GAL-13-treated and untreated cells. *C*, heatmap of 210 differentially expressed genes (DEGs). *Low* expression is shown in *red*, and *high* expression in *green*. *D*, volcano plot of DEGs between control and treated cells. DEGs with *p* < 0.05 and |log2 Fold Change| > 1 are considered significant (*red* points indicate 159 upregulated genes, and *blue* points indicate 230 downregulated genes). *E*, Bubble plot of Gene Ontology (Molecular Function) for DEGs. *F*, scatter plot comparing median read counts between treatment and control conditions, with DEGs highlighted in *red* (upregulated) and *blue* (downregulated).

Although GAL-13 has been primarily studied for its immunomodulatory and placental functions, little is known about its ability to modulate gene expression in cancer cells. Given that GAL-13 is expressed in liver cancer, we used HepG2 cells to explore its potential to activate transcriptional programs in liver cancer cells. This well-characterized cell line retains many differentiated hepatic functions and provides a relevant model for studying the transcriptional response of liver cancer cells to GAL-13 treatment. After validating the ability of GAL-13 to specifically bind to HepG2 cells (Fig. S1), the cells were incubated for 16 h with recombinant human GAL-13 [5 μM], followed by RNA sequencing to assess global transcriptional changes (Fig. 1*A*). The multidimensional scaling analysis revealed a clear segregation between GAL-13–treated and control samples, indicating that GAL-13 exposure profoundly altered the transcriptomic landscape of HepG2 cells (Fig. 1*B*). This separation was highly consistent across biological replicates, confirming the reproducibility of the observed expression patterns. The heatmap further highlighted distinct gene clusters between the two experimental conditions, with a group of transcripts markedly upregulated in response to GAL-13 treatment (Fig. 1*C*). Differential expression analysis identified a subset of genes significantly induced by GAL-13, including *CCL20*, *SERPINE1*, *NR4A1*, *IGFBP1*, and *EGR1*, many of which are associated with inflammatory signaling, cell adhesion, and angiogenic processes. In contrast, a smaller number of genes were significantly downregulated (Fig. 1*D*). Functional enrichment analysis of the upregulated genes revealed significant enrichment in categories related to receptor ligand activity, cytokine activity, and growth factor signaling, suggesting that GAL-13 may promote activation of pathways involved in intercellular communication and tumor-microenvironment interactions (Fig. 1*E*). Finally, a direct comparison of read counts between treated and control samples confirmed a consistent and robust induction of key regulatory genes following GAL-13 exposure (Fig. 1*F*).

**Figure 2:**
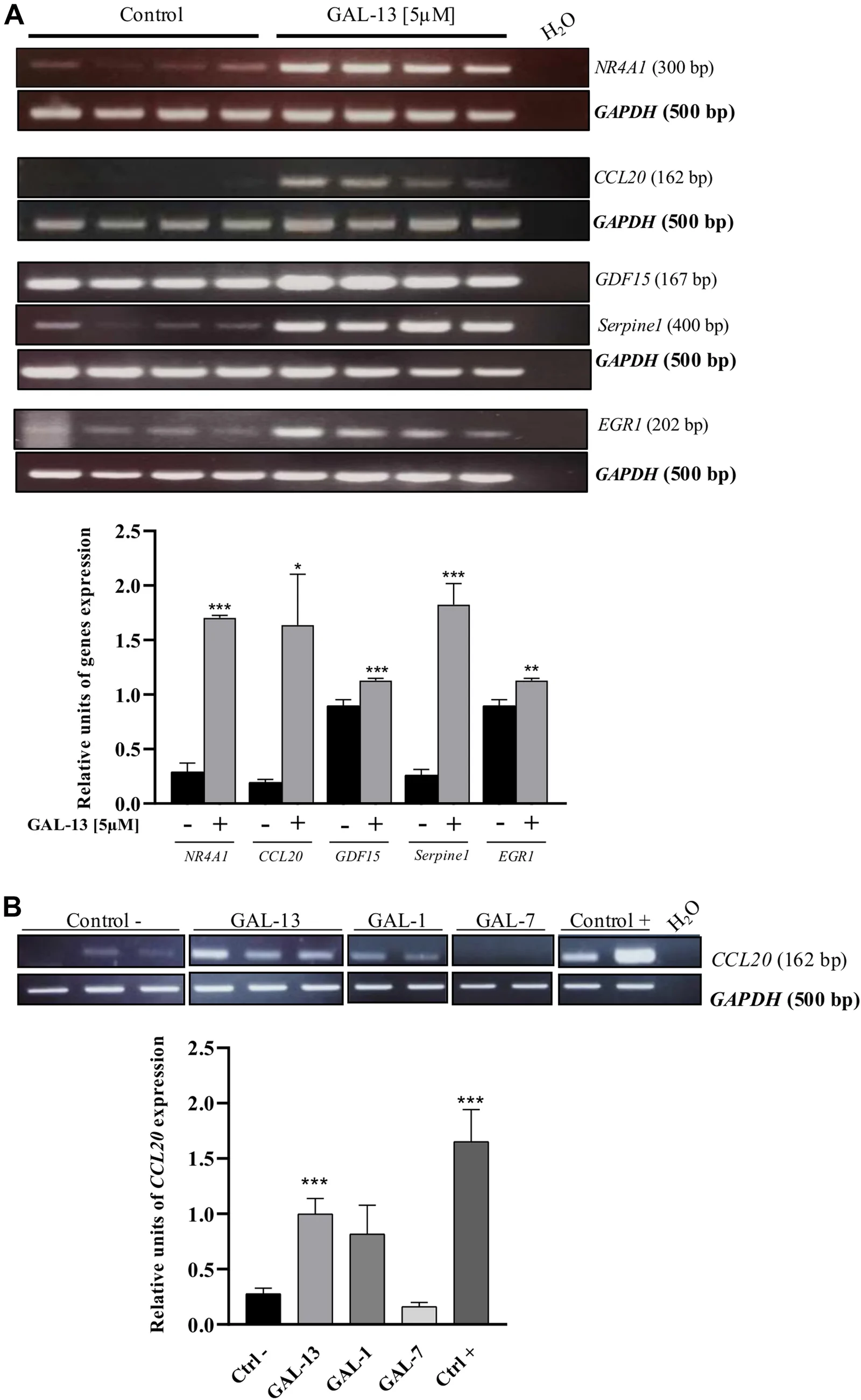
**RT-PCR validation of transcriptomic data.***A*, semi-quantitative agarose gels of gene expression (*NR4A1*, *CCL20*, *GDF15*, *Serpine1*, *EGR1*) in HepG2 cells treated or not with GAL-13 at 5 μM for 8 h. Data were expressed in relative units. ∗*p* < 0.05, *∗∗p* < 0.01, *∗∗∗p* < 0.001. *B*, semi-quantitative agarose gels of *CCL20* gene expression in HepG2 cells treated with 5 μM GAL-13, GAL-7, or GAL-1 for 8 h. The positive control was PMA [200 ng/ml], and all data were compared to the negative control. ∗∗∗*p <* 0.001.

To confirm the transcriptional activity induced by recombinant human GAL-13, the expression of several immediate-early genes associated with stress responses, cytokine regulation, tumor microenvironment remodeling, and inflammation-related processes, including *NR4A1*, *CCL20*, *GDF15*, *Serpine1*, and *EGR1*, was analyzed by RT-PCR (Fig. 2*A*). The mRNA levels of *NR4A1*, *CCL20*, *GDF15*, *Serpine1*, and *EGR1* were increased. Densitometric quantification confirmed significant induction of each target gene relative to the control, with *NR4A1*, *GDF15*, and *Serpine1* showing the most pronounced upregulation. Further experiments focusing on *CCL20*, a chemokine that plays a key role in cancer progression and, in the case of liver cancer, is associated with increased tumor invasiveness and poor clinical outcome, showed that its upregulation was specific to GAL-13 (Fig. 2*B*). Overall, these transcriptomic data demonstrate that recombinant GAL-13 can act as a signaling modulator in HepG2 liver cancer cells, promoting the expression of genes involved in cytokine and growth factor networks, thereby supporting the hypothesis that GAL-13 contributes to the activation of pro-tumoral signaling pathways.

### Generation of GAL-13-binding nanobodies

**Figure 3:**
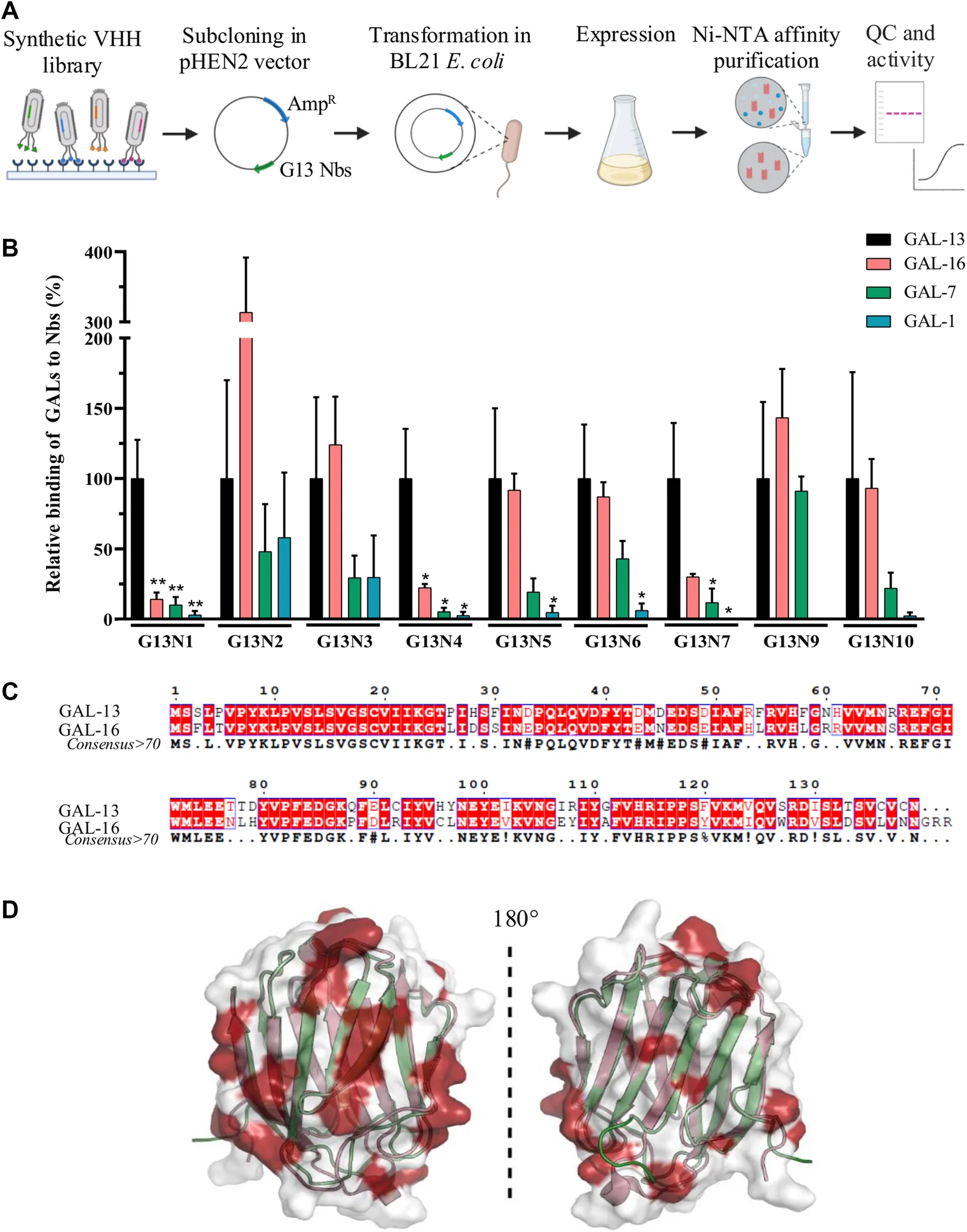
**Generation and characterization of anti-GAL-13 Nbs.***A*, schematic of VHH selection from a synthetic library, production in *E. coli* expression systems, and purification by nickel column chromatography. *B*, ELISA measurements of binding of all Nbs to GAL-1, GAL-7, GAL-13, and GAL-16. Data (mean ± SEM) were standardized to GAL-13. *∗p* < 0.05, *∗∗p* < 0.01. *C*, multiple sequence alignment of GAL-13 and GAL-16 showing 76% sequence identity. Strictly conserved residues are highlighted in *white text* within *red boxes*. The alignment was performed with Clustal Omega and visualized with ESPript 3.0. *D*, overlay of the CRDs of GAL-13 (*purple cartoon*, PDB 5XG7) and GAL-16 (*green cartoon*, PDB 6LJP) illustrates strong structural similarity. Electron density representation highlights surface positions that are conserved (*white surface*) or distinct (*red surface*) between the two galectins.

Given the limited availability of molecular tools to study GAL-13 and its emerging roles in cancer and immune regulation, we sought to generate single-domain camelid nanobodies (Nbs) targeting GAL-13 to develop both research reagents and potential neutralizing candidates. Using a synthetic phage display library, we isolated GAL-13-specific Nbs through iterative rounds of panning and selection, followed by cloning into the pHEN2 vector for expression in *E. coli* BL21 and purification (Fig. 3*A*). A total of 10 clones were obtained. ELISA analyses confirmed that several of the selected clones bound strongly to GAL-13 (Table 1). Several of these clones exhibited partial cross-reactivity with GAL-16, but not with other galectin family members such as GAL-1 or GAL-7 (Fig. 3*B*). Sequence alignment and structural modeling revealed a high degree of amino acid and three-dimensional similarity between GAL-13 and GAL-16, potentially explaining this cross-reactivity (Fig. 3, *C* and *D*). Overall, these Nbs constitute the first generation of molecular tools specifically designed to interrogate GAL-13 biology, offering new opportunities for mechanistic and research applications.

**Table 1:** CDR sequences of the 10 Nbs targeting GAL-13

Nanobody	ELISA ratio	a.a.	MW (Da)	pI	CDR1	CDR2	CDR3
G13N1	34,3	121	13013.47	8.66	FGSNRST	SLSSDPP	PAG---------MRGVME
G13N2	28,7	127	13954.58	9.94	STSRSSG	YRGSGAR	MWQRQIQPG---TRPVWK
G13N3	27,1	124	13748.39	9.78	RGSRWYG	AQHSTRA	GYMSKMG------ERKWK
G13N4	26,5	121	13034.38	8.58	SSYAGSA	SDPDTKA	ISG---------QYPYAR
G13N5	17,7	130	14373.99	9.36	YTSNEDS	RGPSFRT	IRLGRLYRYGDLTPKSAY
G13N6	16,5	121	13249.8	9.26	RTSSLTS	FSGSMFT	DKWMHKPAN---------
G13N7	14,5	121	13431.87	9.26	RTWNSYT	GEPGGIR	DRWP---------NKHDR
G13N8	12,4	127	14035.51	8.58	TTWKQET	RGPNYYP	DEWMGSINA---RHGSGY
G13N9	10,5	124	13369.88	9.26	RGSALTT	GDTGLYV	GKWLG---G---RQADTR
G13N10	9,7	126	13935.4	8.97	R-TSNQT	ARFDEYG	LGWPQKSTW---RAYTAE

### Neutralizing activity of GAL-13-selective nanobodies

**Figure 4:**
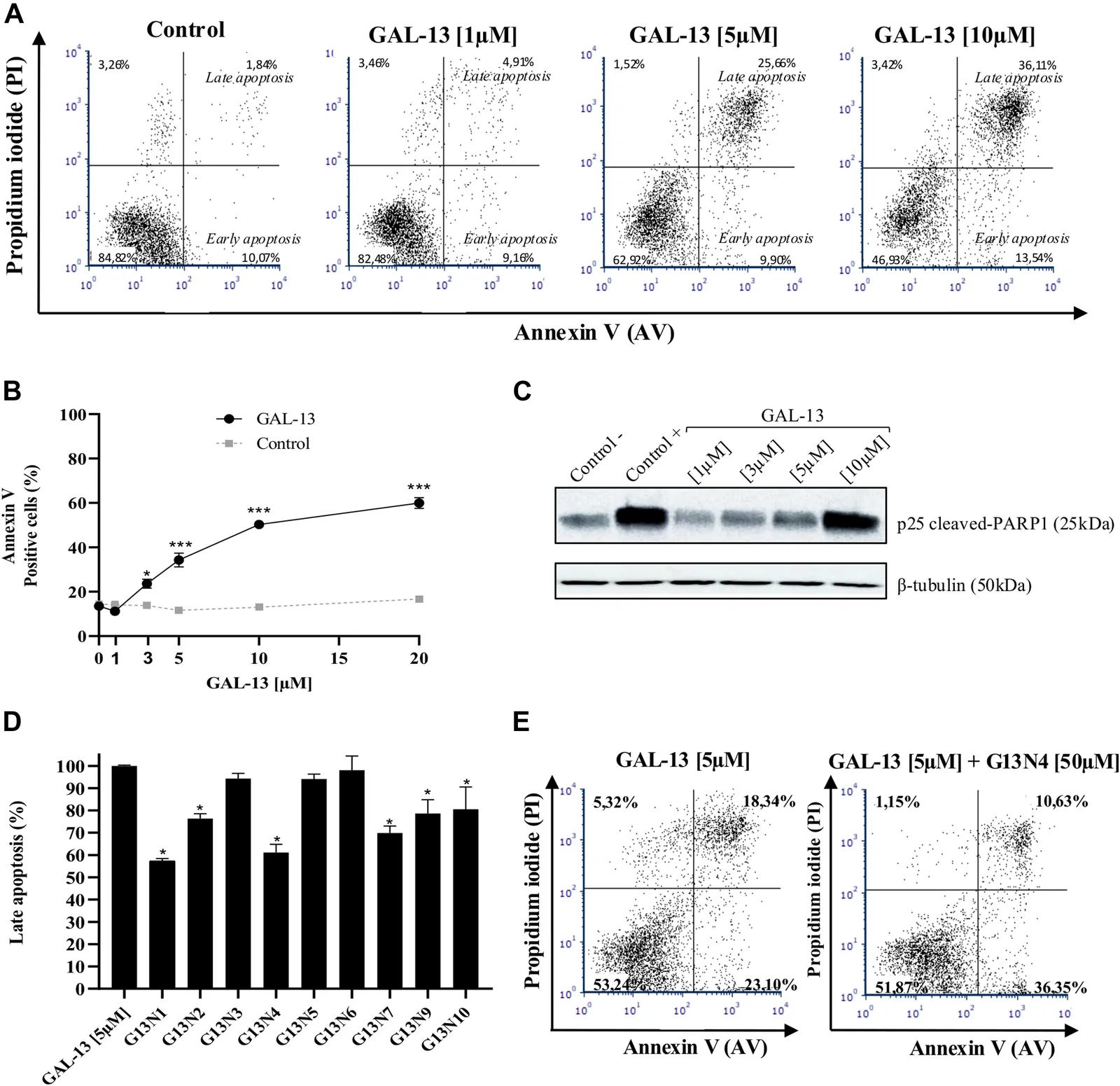
**GAL-13 Nanobodies delay T cell apoptosis induced by GAL-13.***A*, Representative flow cytometry dot plots showing Jurkat T cell apoptosis induced by a dose-response of GAL-13. T cells were incubated with increasing doses of GAL-13 or vehicle control for 16 h and labeled with PI and Annexin V. *B*, dose-response of GAL-13-induced apoptosis in Jurkat T cells. Experiments were performed with three biological replicates (mean values ± SEM). *∗p* < 0.05, *∗∗∗p* < 0.001. *C*, Western blot analysis confirmed the pro-apoptotic effect of GAL-13 by PARP-1 cleavage. The positive control was DMSO. *D*, Jurkat T cells were treated with GAL-13 (5 μM) that had been preincubated for 1 h at 4 °C with individual nanobodies (Nbs, 50 μM) or vehicle. Late apoptosis was quantified 16 h later by flow cytometry. Values were normalized to the GAL-13-alone condition (set to 1 or 100%). Bars show mean ± SEM; the number of independent experiments is provided in Experimental procedures. Statistical comparisons were made *versus* GAL-13 alone; ∗*p* < 0.05. *E*, representative dot plot of late apoptosis delay induced by G13N4.

**Figure 5:**
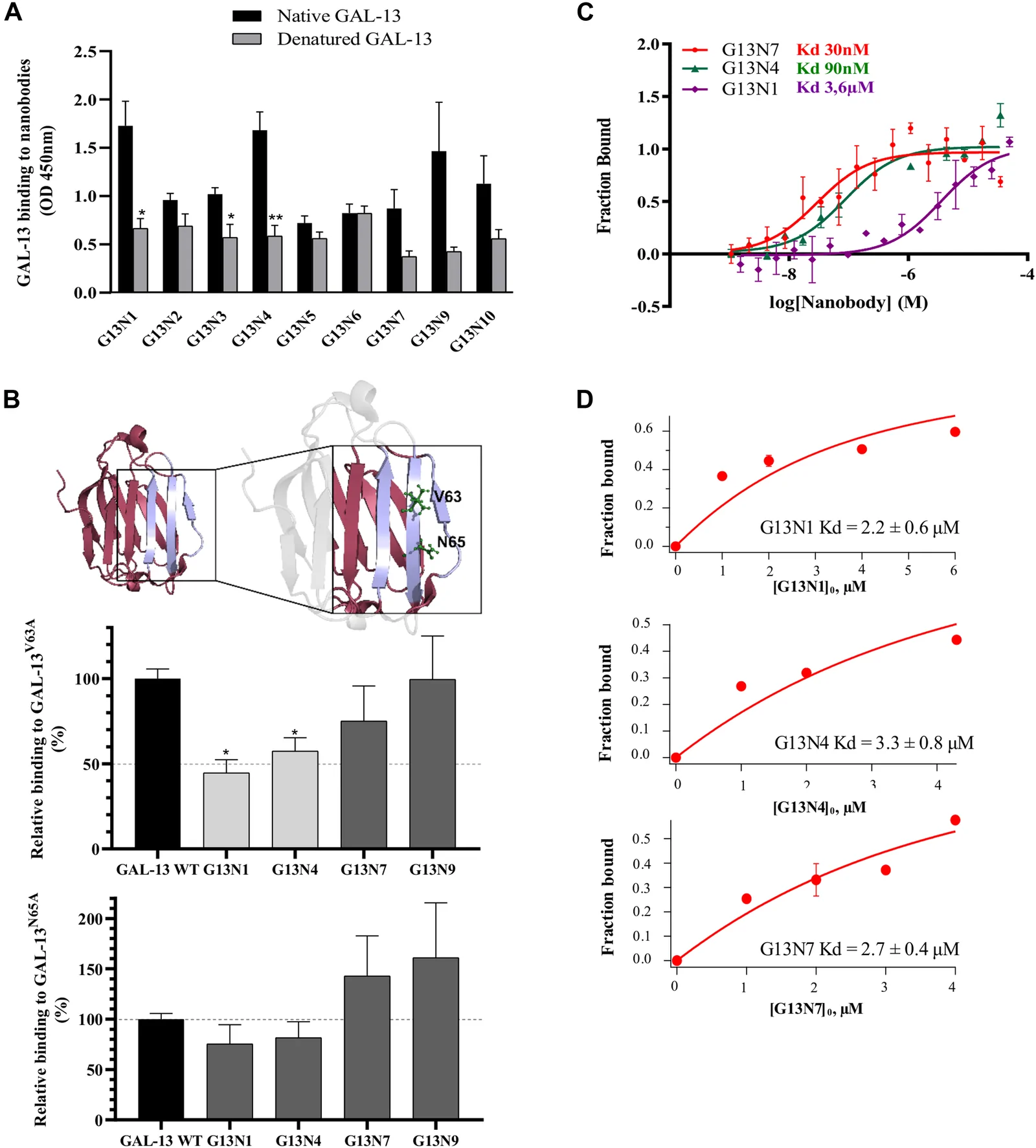
**Anti-GAL-13 nanobodies: characterization and functional modulation *in vitro*.***A*, nanobody sensitivity to bind conformational epitopes on GAL-13 (native GAL-13 in a continuous line and denatured GAL-13 in a dashed line), measured by ELISA. *∗p* < 0.05, *∗∗p* < 0.01. *B*, the GAL-13 CRD is shown as a cartoon, with β-strands forming the canonical galectin GBS highlighted in *light blue*. Residues V63 and N65, located within or near this region, are shown as sticks and were individually substituted by alanine. The nanobody epitopes on GAL-13 are not depicted, as no GAL-13-nanobody co-complex structure is currently available. Binding of G13N1, G13N4, G13N7, and G13N9 to GAL-13 V63A or N65A was measured by ELISA and normalized to binding to WT GAL-13, set to 100%. Reduced binding to a mutant indicates sensitivity to perturbation of the GBS region and suggests either proximity to this region or local conformational/electrostatic coupling. Data are mean ± SEM; ∗*p* < 0.05 *versus* WT GAL-13. *C*, microscale thermophoresis (MST) analysis between GAL-13 and its ligands G13N1 (*purple*), G13N4 (*green*), and G13N7 (*red*). *D*, plot of the fraction of Nbs bound to GAL-13, quantified by SLOMO-nMS.

Because many members of the galectin family are known to induce apoptosis through glycan-dependent mechanisms, we next investigated whether GAL-13 exerts a similar pro-apoptotic effect using Jurkat T cells, a well-established model for assessing galectin-mediated cell death (Fig. 4). Cells were treated with increasing concentrations of recombinant GAL-13 and analyzed by Annexin V/propidium iodide staining and flow cytometry. GAL-13 induced a clear, dose-dependent increase in both early and late apoptotic cell populations compared with untreated controls, reaching maximal activity at [10 μM] (Fig. 4*A*). Quantitative analysis confirmed this trend, and Western blot detection of cleaved PARP-1 further validated activation of apoptotic pathways following GAL-13 exposure (Fig. 4, *B* and *C*). To assess the functional neutralizing capacity of the newly generated Nbs, Jurkat cells were co-treated with GAL-13 and individual anti-GAL-13 Nbs clones. Several Nbs significantly reduced GAL-13-induced apoptosis, with clones G13N1 and G13N4 showing the strongest inhibitory effect (Fig. 4*D*). Consistently, pre-incubation of GAL-13 with G13N4 before cell treatment markedly decreased the proportion of Annexin V-positive cells (Fig. 5*E*). These findings demonstrate that GAL-13 can trigger apoptosis in Jurkat T cells and that specific Nbs binding can neutralize this pro-apoptotic activity, highlighting the potential of these reagents as functional GAL-13 inhibitors.

### Characterization of anti-GAL-13 Nbs

To further characterize the lead anti-GAL-13 Nbs identified for their inhibitory activity, we next examined their binding properties using a combination of biochemical and biophysical assays (Fig. 5). We first examined whether the Nbs can bind native or denatured GAL-13 (Fig. S2). Our results revealed that several Nbs, including G13N1, G13N4, and G13N7, displayed markedly reduced binding to denatured GAL-13, indicating that they recognize conformational epitopes and likely interact with the folded protein surface (Fig. 5*A*). Because the carbohydrate recognition domain (CRD) contains the glycan-binding site (GBS) in galectins, we used two GAL-13 alanine variants, V63A and N65A, located within or near the predicted GBS, as local mutational probes to assess whether binding of selected nanobodies was sensitive to perturbation of this region. This assay does not define the complete nanobody-binding sites on GAL-13. However, reduced binding to a given mutant suggests that the corresponding nanobody epitope is either proximal to the GBS or conformationally/electrostatically coupled to this region. G13N1 and G13N4 showed reduced binding to the V63A and/or N65A variants, whereas G13N7 and G13N9 were largely unaffected, suggesting that G13N1 and G13N4 recognize GBS-proximal or GBS-sensitive conformational epitopes, while G13N7 and G13N9 bind epitopes that are less dependent on this region (Fig. 5*B*), suggesting that the former two nanobodies interact in close proximity to the GBS, whereas the latter target distinct or more distal epitopes. To quantify binding affinities, microscale thermophoresis (MST) was performed, yielding dissociation constants (Kd) in the nanomolar range for G13N7 (≈30 nM) and G13N4 (≈90 nM), while G13N1 bound more weakly (≈3.6 μM) (Fig. 5*C*). These results were further validated by SLOMO-nMS analysis, which confirmed relative differences in binding strengths among the nanobody–GAL-13 complexes (Fig. 5*D*). Together, these findings demonstrate that G13N4 and G13N7 are high-affinity Nbs targeting conformational epitopes on GAL-13, making them strong lead candidates for functional studies and further development of neutralizing reagents.

### Affinity purification and functional validation of recombinant GAL-13

**Figure 6:**
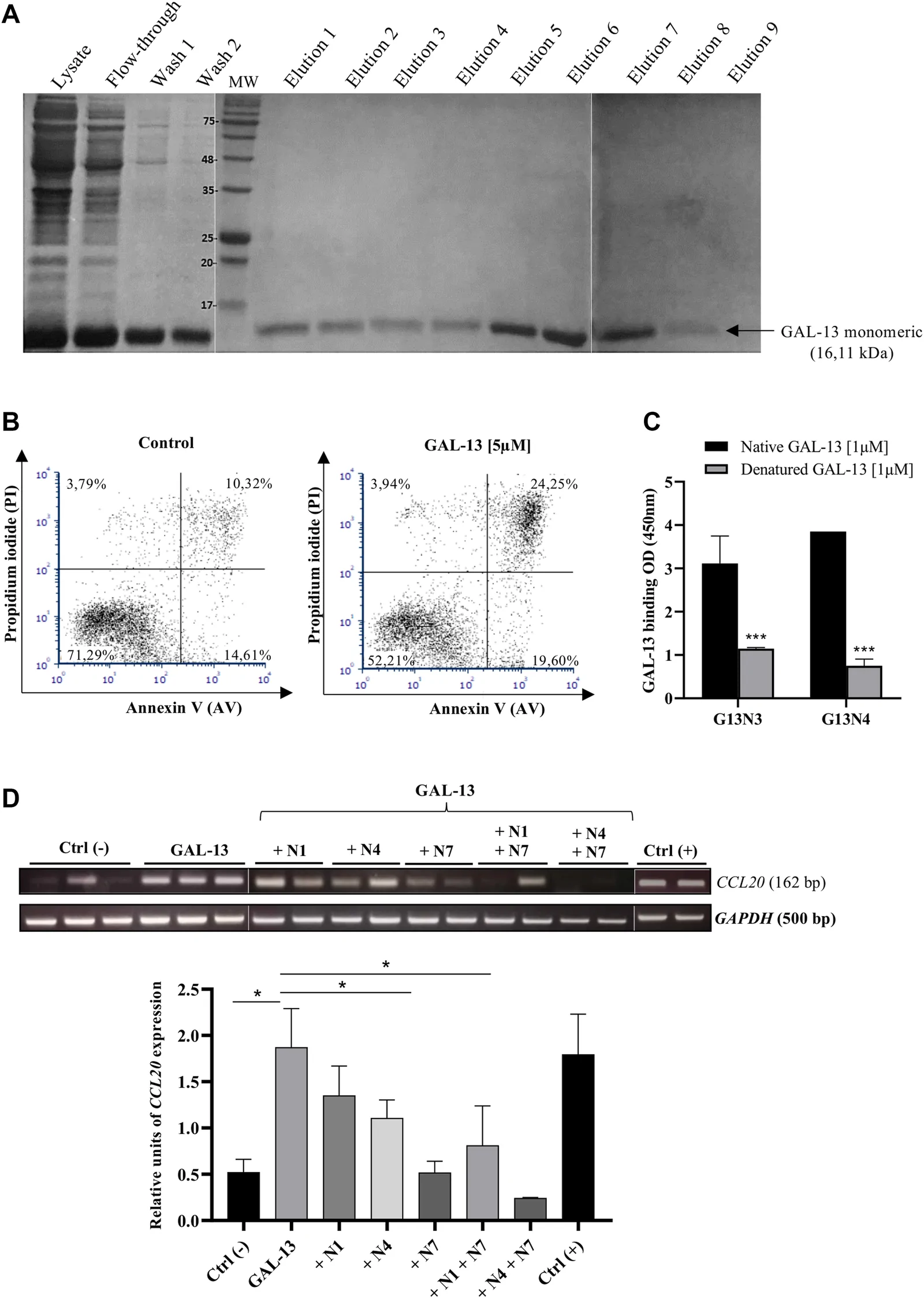
**GAL-13 Nanobodies as research tools.***A*, SDS-PAGE analysis of purified GAL-13 using Nbs coupled to Sepharose beads. The column was washed with Tris-NaCl solution, and GAL-13 was eluted with a low-pH solution. A portion of the first gel containing several successive wash eluates was removed for figure presentation. For presentation, non-adjacent wash lanes from the first gel were removed; breaks are indicated by thin divider lines. All lanes derive from the same experiment but were loaded on multiple acrylamide gels run and processed in parallel under identical conditions. *B* and *C*, biological effects of the newly purified GAL-13, confirmed by induction of Jurkat T cell apoptosis and by its ability to bind Nbs [10 μM]. *D*, validation of the biological effects of Nbs on GAL-13-induced *CCL20* gene expression. Semi-quantitative agarose gel analysis of *CCL20* mRNA expression in HepG2 cells treated with GAL-13 [5 μM] in the presence or absence of Nbs [50 μM] for 8 h. The gel images were rearranged for figure presentation. All lanes originate from the same experiment, and both gels were imaged and processed with identical parameters. The positive control was PMA [200 ng/ml], and all data were compared to the negative control. *∗p <* 0.05.

Production of GAL-13 is considered complex due to its unique structural properties, making it less straightforward to produce than many other galectins. To address these limitations, we established an Nbs-based affinity chromatography strategy to efficiently purify soluble, functional GAL-13. GAL-13 was expressed in *E. coli* BL21(DE3) and purified using an Nb-based affinity column containing G13N1, G13N4, and G13N7 coupled to Sepharose beads. The recombinant protein was efficiently recovered from the bacterial lysate upon low-pH elution (Fig. 6*A*). Flow cytometric analysis showed that affinity-purified GAL-13 retained its proapoptotic activity (Fig. 6*B*) and, upon denaturation, lost its binding to Nbs, attesting to the quality of the purification (Fig. 6*C*). It also retained its ability to induce *CCL20* expression in HepG2 cells, and preincubation of GAL-13 with G13N4 and G13N7 reduced galectin-induced overexpression of *CCL20* (Fig. 6*D*). Our three lead Nbs demonstrated strong methodological value and potential research applications, thereby facilitating future research on GAL-13.

## Discussion

Although GAL-13 was initially characterized for its immunomodulatory roles at the maternal–fetal interface, our understanding of this lectin remains remarkably limited. Most of the current knowledge comes from studies focused on pregnancy physiology, placental immune tolerance, and disorders such as pre-eclampsia (12). Beyond these contexts, the functions of GAL-13 in other normal and disease processes are largely unknown. In the present work, we have addressed several of these limitations by providing novel insights into the biology of GAL-13 and by introducing new research tools to study its physiological and functional roles. We showed that (1) a panel of high-affinity single-domain Nbs targeting GAL-13 was successfully generated using a synthetic phage display library and characterized through detailed biophysical analyses; (2) these Nbs recognized conformational epitopes on the folded GAL-13 protein and enabled an efficient nanobody-based affinity purification method for isolating functional recombinant GAL-13; (3) recombinant GAL-13 was capable of inducing apoptosis in Jurkat T cells in a dose-dependent manner, demonstrating its ability to activate cell-death pathways similar to other galectins; (4) transcriptomic profiling of HepG2 liver cancer cells exposed to recombinant GAL-13 revealed extensive transcriptional reprogramming, with marked upregulation of stress-, cytokine-, and tumor-microenvironment-related genes; and (5) our lead Nbs exhibited functional efficacy by inhibiting GAL-13–induced apoptosis and expression of *CCL20*, underscoring their potential as both mechanistic research tools for neutralizing GAL-13 activity.

For the first time, we have identified a previously unknown function of GAL-13: its ability to induce and modulate the transcriptome of cancer cells. Transcriptomic profiling of HepG2 liver cancer cells exposed to recombinant GAL-13 showed induction of many genes, including stress- and cytokine-related genes such as *NR4A1*, *CCL20*, *GDF15*, *EGR1*, and *SERPINE1*. These genes are generally triggered by cellular stress, hypoxia, or inflammation and play important roles in tumor progression. *NR4A1*, for example, has been shown to promote cell survival and resistance to apoptosis (22), while *CCL20* is involved in the recruitment of immune cells, contributing to establishing a pro-inflammatory and metastatic tumor microenvironment (23, 24), supporting the hypothesis that GAL-13 has an impact on the tumor microenvironment. In the case of *GDF15*, it has been shown to favor immune tolerance and therapeutic resistance (25, 26). Finally, *EGR1* and *SERPINE1* have been shown to facilitate migration, invasion, and metastasis in multiple ways (27, 28, 29). Collectively, these genes form a signature of stress, invasion, and immunosuppression characteristic of aggressive cancers such as triple-negative breast cancer. Functional enrichment analyses further identified pathways related to cytokine and growth factor signaling, suggesting that GAL-13 may influence interactions within the tumor microenvironment. These findings support previous *in silico* studies indicating that this galectin may be involved in cancer, along with the recent discovery of its role in ferroptosis (30). Overall, our findings expand the biological understanding of GAL-13 beyond its known placental functions and suggest a potential glyco-immune checkpoint role with implications for cancer progression. Nonetheless, this study was limited to a single *in vitro* model, and further research is required to validate these transcriptomic effects in additional cancer cell lines and in more physiologically relevant systems. Future studies should also incorporate *in vivo* models, including genetically engineered mouse models and disease-relevant *in vivo* systems in which Nbs can serve as mechanistic probes and therapeutics. In this latter case, given the flexibility of Nbs, one can envision the development of neutralizing and bispecific Nbs to target pathophysiological processes such as angiogenesis, immune remodeling, invasion, or placental development outcomes. These studies will help better determine the clinical relevance of GAL-13 and its potential as an actionable target or biomarker in cancer.

We developed high-affinity single-domain Nbs targeting native GAL-13 (Fig. S3). Developing Nbs against GAL-13 offers several advantages for both research applications. Given their small size, ease of production in bacterial expression systems, and capacity to recognize unique epitopes, Nbs are outstanding research tools that enable precise detection, functional analysis, and modulation of target proteins (31, 32). They are also ideally suited for *in vivo* imaging and, as in this case, could help examine GAL-13 expression in normal tissues and in primary tumors that express galectins. We have shown this in our recent studies using Nbs against GAL-1 and GAL-7 (33, 34). The nanobody-based affinity chromatography strategy proved particularly effective for producing soluble, properly folded, and biologically active GAL-13, thereby overcoming a longstanding challenge in the field—its instability and poor yield in recombinant expression systems (35). From a research perspective, the Nbs could be very useful for neutralizing the biological activity of GAL-13, whether in its developmental role or during tumorigenesis. For example, our results showing that these Nbs can block GAL-13-induced *CCL20* gene expression suggest that they could help reverse immune suppression, restore anti-tumor immune activity, and complement existing immunotherapies. Overall, they represent valuable tools to study the distribution, molecular interactions, and biological functions of GAL-13 in cancer and may pave the way for the development of new targeted immunotherapies.

Our results also reveal that several Nbs cross-react with GAL-16, a close structural homolog of GAL-13. Sequence and structural modeling indicate a high degree of similarity between the two proteins, likely explaining the observed cross-reactivity. This finding highlights their evolutionary relatedness. GAL-16 is encoded by the *LGALS16* gene, located within a cluster of placental galectins that also includes *LGALS13* (11). Like GAL-13, its expression is primarily placental, where it may contribute to immune tolerance at the maternal–fetal interface by inducing T cell apoptosis (36, 37). However, GAL-16 is also found in several other tissues, including the brain and retina, as well as in various cancer cell lines (37). As with GAL-13, the physiological and pathological roles of GAL-16 remain poorly understood due to the lack of specific antibodies, underscoring the need for further molecular and functional research. These new Nbs will thus be particularly valuable for future studies aimed at comparing the functional activity of GAL-13 with that of GAL-16. By pairing functional assays with phylogenetic analyses of these two structurally conserved paralogs, we could further trace whether new functions have emerged after gene duplication and subsequent changes in glycan specificity and affinity, oligomerization, or receptor engagement. It will also help us better determine whether the act redundantly, where they diverge, and how those patterns map onto disease and, from a fundamental perspective, how a conserved galectin scaffold can acquire new context-specific roles through fine-scale sequence tweaks. However, it will be necessary to develop more specific Nbs to better distinguish the roles and expression of these two galectins across different contexts. For example, we might use clone G13N2, which shows a higher affinity for GAL-16 than GAL-13, to create a more specific antibody by combining structural approaches and guided selection. Comparative structural analysis of GAL-13 and GAL-16 enables the identification of distinctive residues to target for strengthening specific interactions with GAL-16 or through directed or random mutagenesis in the CDR3 region, responsible for antigen binding, during phage selection, along with negative selection steps against GAL-13. The cross-reactivities observed with several Nbs raise an important methodological consideration: research on GAL-13 (and other tandem-repeat galectins) must carefully evaluate reagent specificity. Indeed, this approach is ideally suited for (*i*) preparative purification of native, active GAL-13/-16 from tissues or cells; (*ii*) pull-down assays to discover currently unknown endogenous ligands; and (*iii*) immunoprecipitation/co-IP to define macromolecular complexe. It also offers a robust alternative to epitope tags (His, c-Myc, FLAG), which require transfection and can sometimes perturb protein folding, interactions, charge/solubility, or introduce assay artifacts.

In conclusion, our study makes both conceptual and methodological contributions to GAL-13 research. Future efforts should focus on analyzing receptor interactions and glycan-binding dependencies that drive these effects, as well as on clarifying the relationship between GAL-13 and structurally related proteins, such as GAL-16. Expanding the range of validated reagents, combined with multi-omics and *in vivo* approaches, will be essential to fully understand the biological importance of this understudied lectin family.

## Experimental procedures

### Expression and purification of human recombinant GAL-13 wild-type

Codon-optimized vectors encoding human galectins (GAL) were inserted into pET-22b(+) and transformed into *E. Coli* BL21 (DE3). For protein expression, cells were grown in LB-Miller broth, induced with 1 mM IPTG, and grown for 4 h at 37 °C. Human GAL-13 was purified by affinity chromatography using mannose-agarose beads (Sigma-Aldrich) and eluted with a NaCl gradient in a 25 mM Tris buffer. Human GAL-16 was recovered from inclusion bodies and refolded using 10% [v/v] of glycerol and [1M] 2-methyl-2,4-pentanediol. Other galectins were purified using lactose-agarose beads and eluted with a 150 mM lactose solution. GAL-13 was dialyzed and stored in the following buffer: Tris [25 mM], NaCl [150 mM], pH 8.

### Production and purification of human recombinant GAL-13 mutants

Protein Expression and Purification GAL-13 and variants were expressed in *E. coli* BL21(DE3) using pET-22b(+). Cultures were induced at an OD600 of 0.6 with 0.5 mM IPTG and grown for 16 h at 16 °C. Cells were harvested, lysed by sonication, and the supernatant was dialyzed (3 kDa MWCO) against Bis-TRIS (20 mM), pH 7.0. The sample was filtered (0.22 μm) and purified using a HiTrap Q column (GE Healthcare) with a linear NaCl gradient. Final purification was performed *via* size-exclusion chromatography on a Sephacryl S-300 HR column equilibrated in Bis-TRIS [20 mM], pH 7.0, NaCl [100 mM]. Purity was confirmed by SDS-PAGE.

### Expression and purification of nanobodies

GAL-13-specific Nbs were generated by Hybrigenics (Évry-Courcouronnes, France). Briefly, a synthetic library was screened against the preparation of biotinylated recombinant full-length human GAL-13 protein. After three rounds of selection, VHH clones were randomly selected and tested in a non-absorbed phage ELISA assay using avidin-coated plates and biotinylated GAL-13 antigen for cross-validation. Twelve clones were selected based on their amino acid sequences, and their cDNA was cloned into the pHEN2 expression vector, as described by Moutel *et al.* (38). Competent *E. coli* BL21(DE3) bacterial cells were transformed with the various pHEN2 constructs, and expression of Nbs was induced as described (Fig. S1). Nbs were purified by standard immobilized-metal affinity chromatography using pre-packed Cytiva His GraviTrap columns (Sigma-Aldrich). Affinity-purified Nbs were dialyzed against PBS to remove imidazole.

### Cell lines

The Jurkat cell line was maintained in RPMI 1640 medium supplemented with 10% [v/v] fetal bovine serum (FBS), 2 mmol/L L-glutamine, 10 mM HEPES buffer, and 1 mM sodium pyruvate, at 37 °C and 5% CO_2_. The HepG2 cell line was maintained in DMEM containing 4.5 g/L glucose, supplemented with 10% [v/v] FBS, at 37 °C and 5% CO_2_. All cell culture products were purchased from Wisent.

### Bulk RNA-sequencing

HepG2 cells were plated in 24-well plates and grown to 80% confluence. Cells were treated with [5 μM] of recombinant GAL-13 or vehicle and incubated for 8 h at 37 °C. RNA purification was performed using the RNeasy Mini Kit (Qiagen). Library preparation and high-throughput sequencing were conducted by Génome Québec using the Illumina NovaSeq 6000 system. Raw data and bioinformatics analyses were performed at the Bioinformatics Core Facility of the Montreal Clinical Research Institute. The quality of the raw reads was assessed using FASTQC v0.11.8, and no trimming was deemed necessary. Reads were aligned to the GRCh38 (release 108) Ensembl reference genome using STAR v2.7.6a, resulting in a mean of 94% uniquely mapped reads. Raw counts were calculated with FeatureCounts v2.1.0 based on the GRCh38 Ensembl reference genome (release 108). The raw sequencing data generated in this study have been deposited in the NCBI Sequence Read Archive (SRA) under the BioProject accession number PRJNA1436955. The complete R code used for differential expression analyses (DESeq2 processing, statistical testing, and data visualization) is publicly available on GitHub at https://github.com/FannyFronton/RNASeq_Breast_cancer.

### RT-PCR analysis

To validate the expression of specific genes induced by GAL-13 in HepG2 cells, total RNA extraction was performed using RNeasy mini kit (Qiagen) and reverse transcription using M-MuLV reverse transcriptase (New England Biolabs). RT-PCR analysis was carried using the following primers: *CCL20* forward 5′- CTGGCTGCTTTGATGTCAGT-3′ and reverse 5′-AGCATTGATGTCACAGCCTTC-3′ (162 bp); *Serpine*-*1* forward 5′-TGCTGGTGAATGCCCTCTACT-3′ and reverse 5′-CGGTCATTCCCAGGTTCTCTA-3′ (399 bp); *GDF15* forward 5′-CGGTGAATGGCTCTCAGATG-3′ and reverse 5′-CAGGTCCTCGTAGCGTTTCC-3′ (167 bp); *EGR1* forward 5′-GACCGCAGAGTCTTTTCCTG-3′ and reverse 5′-TGGGTTGGTCATGCTCACTA-3′ (202 bp); *NR4A1* forward 5′-CAGCTTGCTTGTCGATGTC-3′ and reverse 5′-CATTGACAAGATCTTCATGGACAC-3′ (300 bp); *GAPDH* forward 5′-CAGCTTGCTTGTCGATGTC -3′ and reverse 5′-GTGTCCATGAAGATCTTGTAATG -3′ (500 bp). Band intensity was measured using ImageJ, and the expression levels of the genes of interest were normalized to *GAPDH*.

### Cell apoptosis

250,000 Jurkat cells were exposed overnight with an increasing dose of GAL-13 [1, 3, 5, 10, and 20 μM] or co-treated with [3 μM] of GAL-13 and [30 μM] of each nanobody. GAL-13 and nanobodies were preincubated together 1 h at 4 °C before cell treatment. Cells were co-labeled with FITC-Annexin V (cat.: 640906; Biolegend) and propidium iodide (PI) (cat.: P4170; Sigma) staining and analyzed using BD FACSalibur flow cytometry. All experiments were performed in three biological replicates, and 5000 events were recorded per sample.

### Western blot analysis

Jurkat cells were incubated overnight with increasing concentrations of GAL-13 in RPMI containing 1% [v/v] FBS at 37 °C. Cells were lysed in RIPA buffer with phenylmethylsulfonyl fluoride (PMSF) as a protease inhibitor for 30 min at 4 °C. Lysates were centrifuged at 15,000*g* for 15 min at 4 °C. Protein lysates were resolved by SDS-PAGE and transferred to PVDF membranes (Bio-Rad) by electroblotting at 100 V for 1 h. PVDF membranes were blocked using PBS buffer with 5% [w/v] nonfat dry milk and 0.1% [v/v] Tween-20. The primary antibodies, Human anti-cleaved PARP1 clone E51 (cat.: ab32064, Abcam) and anti-β-tubulin (cat.: 2128; Cell Signaling Technology), were incubated overnight at 4 °C. After washing, membranes were incubated with HRP-conjugated secondary antibodies for 1 h at RT. Detection was performed using enhanced chemiluminescence Western Blotting Detection Reagents (Cytiva) and ImageQuant LAS 500.

### ELISA assays

Each GAL-13 (native, denatured, or mutant) were immobilized overnight at [0,1 μM] at 4 °C on a flat bottom 96-well polystyrene plate (Costar). After washing with PBS, wells were blocked with PBS containing 10% [w/v] BSA and 0,05% [v/v] Tween 20 for 2 h at RT. Following washes, nanobodies [1 μM] were added to wells for 1 h at RT. Nanobody binding was detected using a goat anti-His-tag polyclonal antibody (Bio-Rad) (1:1000) incubated for 1 h at RT, followed by an anti-goat HRP conjugate (Promega) (1:5000) for 1 h at RT. The colorimetric assay was carried out with 3,3′,5,5′-Tetramethylbenzidine (TMB) according to the manufacturer's recommendations (Sigma-Aldrich). The reaction was stopped after 30 min with 0.16 M sulfuric acid. Absorbance at 450 nm was measured using a Tecan plate reader. For specificity studies, Nbs [1 μM] were immobilized overnight at 4 °C, following washes and blocking steps, and different human galectins were added at [0, 1 μM] for 1 h at RT. Detection of GAL-13 and GAL-16 were revealed using a mouse anti-GAL-13 monoclonal antibody (cat.: ab218411: Abcam) (1:1000), GAL-7 using (cat.: AF1339; R&D Systems) (1:1000) and GAL-1 using (cat.: 60223-1; ProteinTech) (1:1000).

### Microscale thermophoresis

Data was acquired with a Monolith NT.115 Pico instrument (NanoTemper Technologies GmbH, Munich, Germany). Following the manufacturer’s protocol, GAL-13 was labelled with RED-NHS second generation using a Monolith NT.115 Protein Labeling Kit Cy5 labelling (Nanotemper Technologies). The excessive dye was separated using the provided column, and the proteins were eluted in [20 mM] Tris-HCl pH 8, [150 mM] NaCl, supplemented with 0.05% [v/v] Tween 20. Each binding assay experiment consisted of 16 two-fold serial dilutions loaded into NT.115 MST capillaries. MST was induced by a 21 s infrared laser (IR-laser) activation, and all experiments were performed in triplicate.

### Slow-mixing-mode native mass spectrometry (SLOMO-nMS)

The SLOMO-nMS binding experiments were performed using a Q Exactive (Classic) Orbitrap mass spectrometer (Thermo Fisher Scientific). The instrument was equipped with a modified nanoflow ESI (nanoESI) source. All measurements were conducted in positive mode using standard uncoated nanoESI emitters (ESIU-001, AB Glycomics) by applying a voltage of approximately 0.6 to 0.8 kV to a platinum wire inserted inside the tip and in contact with the solution. Two solutions were loaded, in a layered fashion, into the emitter (39). Solution 1 was an ammonium acetate solution (200 mM, pH 7.4) of GAL-13 [2 μM] and Nb [0.5–4.2 μM], and Solution 2 had the same composition, with the exception of a higher Nb concentration [30–50 μM]. Mixing of the solutions (as a result of diffusion, as well as electroosmotic and electrophoretic flow) produces changes in analyte concentrations (40). However, because mixing is slow, the equilibrium distribution of GAL-13, Nb and the GAL-13-Nb complex (Equation 1) in Solution is initially unaffected, allowing the apparent abundance ratio (*R*_app_, Equation 2) to be measured at known solution composition. Upon the onset of mixing, the binding equilibrium will shift due to changes in concentration. Under the conditions used in the present work here, mixing will cause an increase in the concentration of GAL-13-Nb complex, leading to an increase in *R*_*app*_:

(1) GAL−13−Nb⇌GAL−13+Nb

(2) $$R_{\text{app}}=\frac{Ab\left(\text{GAL}-13-\text{Nb}\right)}{Ab\left(\text{GAL}-13\right)}\propto \frac{\left[\text{GAL}-13-\text{Nb}\right]}{\left[\text{GAL}-13\right]}=R$$

where *Ab*(GAL-13-Nb) and *Ab*(GAL-13) are the total abundances (sum of ion signals) of GAL-13-Nb and GAL-13, respectively. The *Ab* of GAL-13 and GAL-13-Nb complex ions and are related to their solution concentrations through their response factors (*RF*s), Equation 3a and b:

(3a) $$Ab\left(\text{GAL}-13\right)=RF_{P}\left[\text{GAL}-13\right]$$

(3b) $$Ab\left(\text{GAL}-13-\text{Nb}\right)=RF_{\text{PL}}\left[\text{GAL}-13-\text{Nb}\right]$$

The relative response factor for GAL-13 and GAL-13-Nb (*RF*_GAL-13/GAL-13-Nb_) is the ratio of *RF*_GAL-13_ to *RF*_GAL-13-Nb_ (Equation 3c):

(3c) $$RF_{\text{GAL}-13/\text{GAL}-13-\text{Nb}}=\frac{RF_{\text{GAL}-13}}{RF_{\text{GAL}-13-\text{Nb}}}=\frac{Ab\left(\text{GAL}-13\right)\left[\text{GAL}-13-\text{Nb}\right]}{Ab\left(\text{GAL}-13-\text{Nb}\right)\left[\text{GAL}-13\right]}$$

The magnitude of *RF*_GAL-13/GAL-13-Nb_ can be found from the ratio of the absolute change in *Ab* between two (acquisition) time points, under conditions where the concentrations are changing, which corresponds to the *RF*_GAL-13/GAL-13-Nb_, Equation 4a,b:

(4a) $$\frac{\left|Ab_{t2}\left(\text{GAL}-13\right)-Ab_{t1}\left(\text{GAL}-13\right)\right|}{\left|Ab_{t2}\left(\text{GAL}-13-\text{Nb}\right)-Ab_{t1}\left(\text{GAL}-13-\text{Nb}\right)\right|}=\frac{\Delta Ab\left(\text{GAL}-13\right)}{\Delta Ab\left(\text{GAL}-13-\text{Nb}\right)}$$

(4b) $$\Delta Ab\left(\text{GAL}-13\right)/\Delta Ab\left(\text{GAL}-13-\text{Nb}\right)=RF_{\text{GAL}-13/\text{GAL}-13-\text{Nb}}$$

where *Ab*_*t1*_(GAL-13) and *Ab*_*t2*_(GAL-13) are the *Ab* measured for GAL-13 at time points *t*_*1*_ and *t*_*2*_, respectively, and *Ab*_*t1*_(GAL-13-Nb) and *Ab*_*t2*_(GAL-13-Nb) are corresponding values for GAL-13-Nb. The *K*_d_ value was obtained by fitting Equation 5 to the *RF* corrected fraction of GAL-13 bound by Nb (*R*/(*R*+1)):

(5) $$\frac{R}{R+1}=\frac{{\left[\text{GAL}-13\right]}_{0}+{\left[\text{Nb}\right]}_{0}+K_{d}-\sqrt{{\left(K_{d}-{\left[\text{Nb}\right]}_{0}+{\left[\text{GAL}-13\right]}_{0}\right)}^{2}+4K_{d}{\left[\text{Nb}\right]}_{0}}}{2{\left[\text{GAL}-13\right]}_{0}}$$

### Cell binding assays

Recombinant GAL-13 was labeled with fluorescein isothiocyanate (FITC) as described (41). FITC-labeled GAL-13 was purified using a PD-10 Sepharose column (GE Healthcare) and eluted with PBS. HepG2 cells were detached by [5 μM] EDTA and incubated for 30 min at 4 °C with several concentrations of FITC-GAL-13 or free FITC. Cell surface labeling was detected using a FACSCalibur flow cytometer (BD Biosciences). Specificity of GAL-13 binding was performed by preincubating cold-GAL-13 for 30 min before incubation with FITC-GAL-13 and FITC-GAL-1.

### Immunoaffinity purification of GAL-13

Nbs were immobilized on a cyanogen bromide-activated Sepharose 4 fast flow (Cytivia), as described (42). Recombinant GAL-13 was produced in the same bacterial expression system described previously and purified by Nbs affinity chromatography. Recombinant GAL-13 was eluted using Glycine buffer [200 mM], pH 2.8, and neutralized in Tris-HCl [3M], pH 8.8. GAL-13 was dialyzed and stored in the following buffer: Tris [25 mM], NaCl [150 mM], pH 8.

### Circular dichroism analysis

The CD spectra were measured on a Jasco J-815 spectropolarimeter equipped with a Peltier thermostatic cell holder. The folding of GAL-13 was assessed after a heat denaturation step at 95 °C. The measurements were performed at 20 °C using a 0.1-cm path-length cell in [50 mM] Tris and [150 mM] NaCl. Far-UV CD spectra were monitored from 200 to 250 nm using GAL-13 at [20 μM]. Baselines were corrected by subtracting the buffer contribution.

### Statistical analysis

Results are shown as mean ± SEM. Statistical analysis was performed using GraphPad Prism version 8.0. An unpaired *t* test was performed to compare two experimental groups. Comparisons were performed using the one-way ANOVA followed by Dunnett’s multiple comparisons test or the two-way ANOVA followed by Sidak’s multiple comparisons test. *p*-value < 0.05 was considered statistically significant.

## Data availability

The data generated in this study are available within the article and its supplementary data files.

## Supporting information

This article contains supporting information.

## Conflict of interest

D. C., N. D., and Y. S. P. are co-inventors on patent applications filed by the Institut National de la Recherche Scientifique (INRS) related to this work, all of which concern the use of nanobodies and minibodies to inhibit biological, physiological, and/or pathological processes involving GAL-7 and GAL-1. All other authors have no competing interests to declare.
