# Supplementary material for: Nanobody-based characterization of Galectin-13 reveals immunomodulatory and tumor-promoting functions
Source: J Biol Chem. 2026 Aug 19;302(10):113467. doi: 10.1016/j.jbc.2026.113467 (PMC13597204; doi:10.1016/j.jbc.2026.113467)
Supplement: Supplementary file 1 [file mmc1.pdf]

Supporting Information

Nanobody-Based Characterization of Galectin-13 Reveals Immunomodulatory and Tumor-Promoting Functions

Camille Fuselier[1], Philippine Granger Joly de Boissel[1], Rita Nehmé[1], Alex Paré[1], Fanny Fronton[1], Sammy Ajarrag[1], Duong Bui[2], Marlène Fortier[1], Alyssa Dumoulin[1], John Klassen[2], Nicolas Doucet[1], David Chatenet[1] and Yves St-Pierre[1,*].

INRS - Centre Armand-Frappier - Santé Biotechnologie, Laval, QC H7V 1B7, Canada.
2) Department of Chemistry, University of Alberta, Edmonton, Alberta T6G 2G2, Canada.

*Corresponding Author: Yves St-Pierre, INRS - Centre Armand-Frappier-Santé Biotechnologie, 531 Boul. des Prairies, Laval, QC H7V 1B7, Canada. Phone: 450-687-5010 ; Fax: 450-686-5501 ; E-mail: yves.st-pierre@inrs.ca.

Table of Content

Figure S1. GAL-13 binds specifically to hepatocellular cancer cells.

Figure S2. Structural and functional characterization of recombinant GAL-13.

Figure S3. Hit-to-Lead process selecting Nbs targeting GAL-13. 7

Figure S1

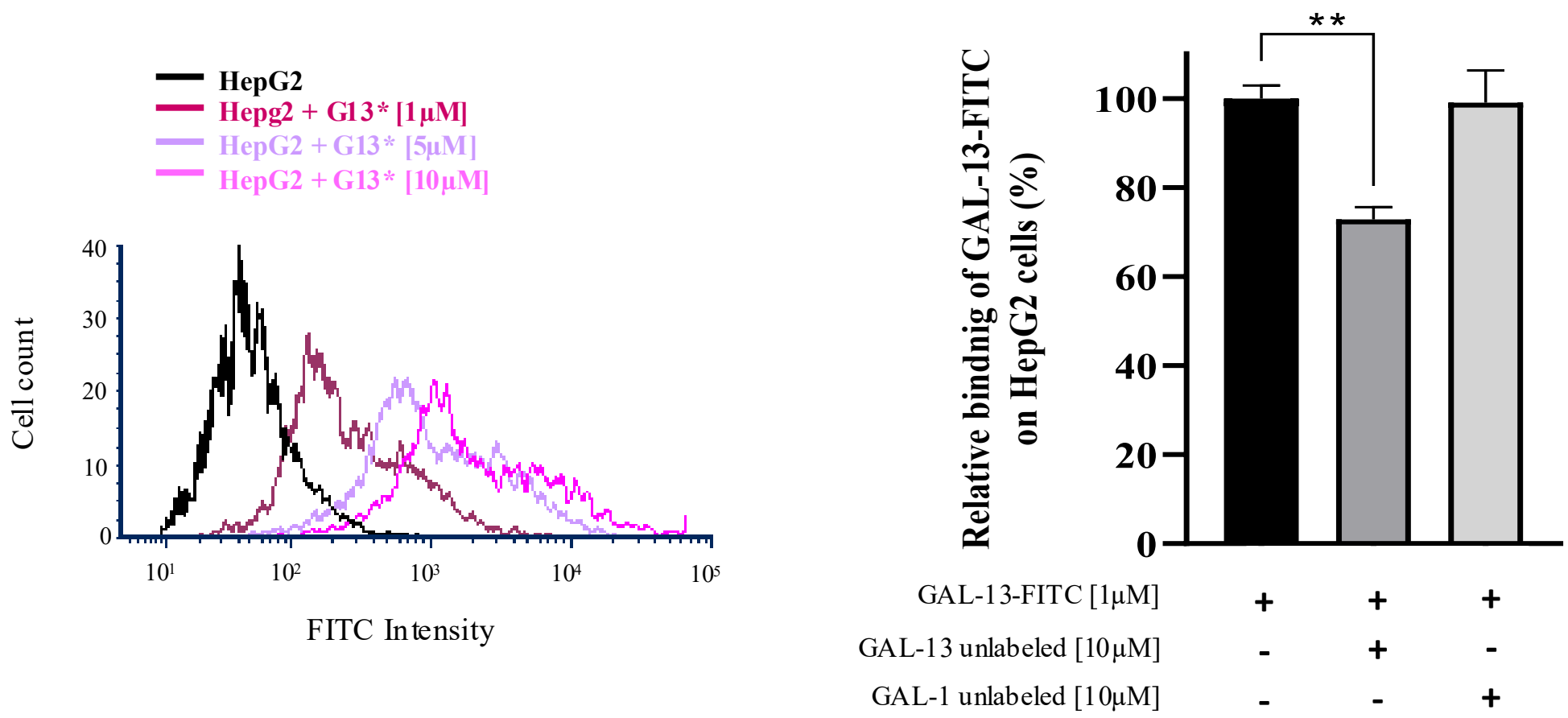

Figure S1. GAL-13 binds specifically hepatocellular cancer cells. (A) Binding profile of an increasing concentration of FITC-GAL-13 on HepG2 cells, detected by flow cytometry. **(B)** Specificity of GAL-13 interaction with cancer cells confirmed by the addition of cold-GAL-13 on HepG2 cells before labeled-galectins. Data were normalized to the FITC-GAL-13 condition. $^{**}P < 0.01$.

Figure S2

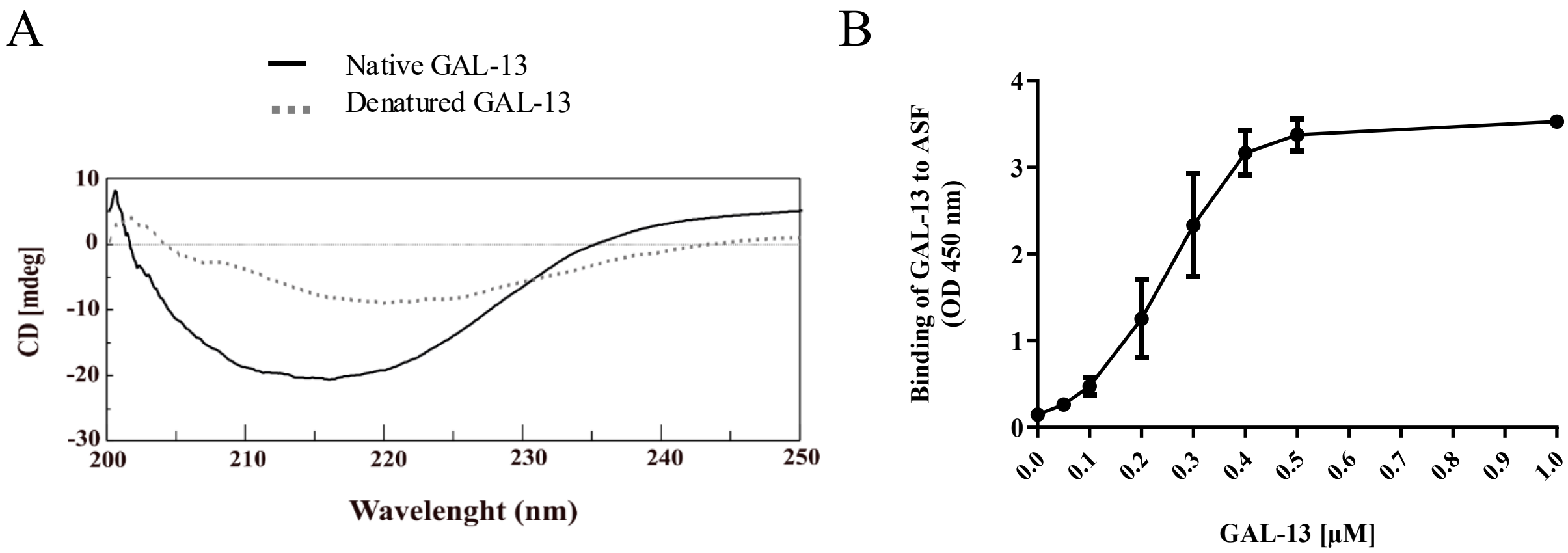

Figure S2. Structural and functional characterization of recombinant GAL-13. (A) Circular dichroism spectra illustrating the folding state of recombinant native GAL-13 (solid line) and heat-denatured GAL-13 (dashed line). The native protein exhibits a pronounced negative band around 215 nm, indicative of a β-sheet–rich secondary structure, whereas upon heat denaturation, this band shifts to longer wavelengths (220–225 nm), reflecting a loss of native folding. **(B)** Binding of increasing concentrations of GAL-13 to immobilized ASF [10 µg/mL], as measured by ELISA. The dose-dependent interaction observed confirms the functional integrity of the GAL-13 carbohydrate recognition domain (GBS).

Figure S3

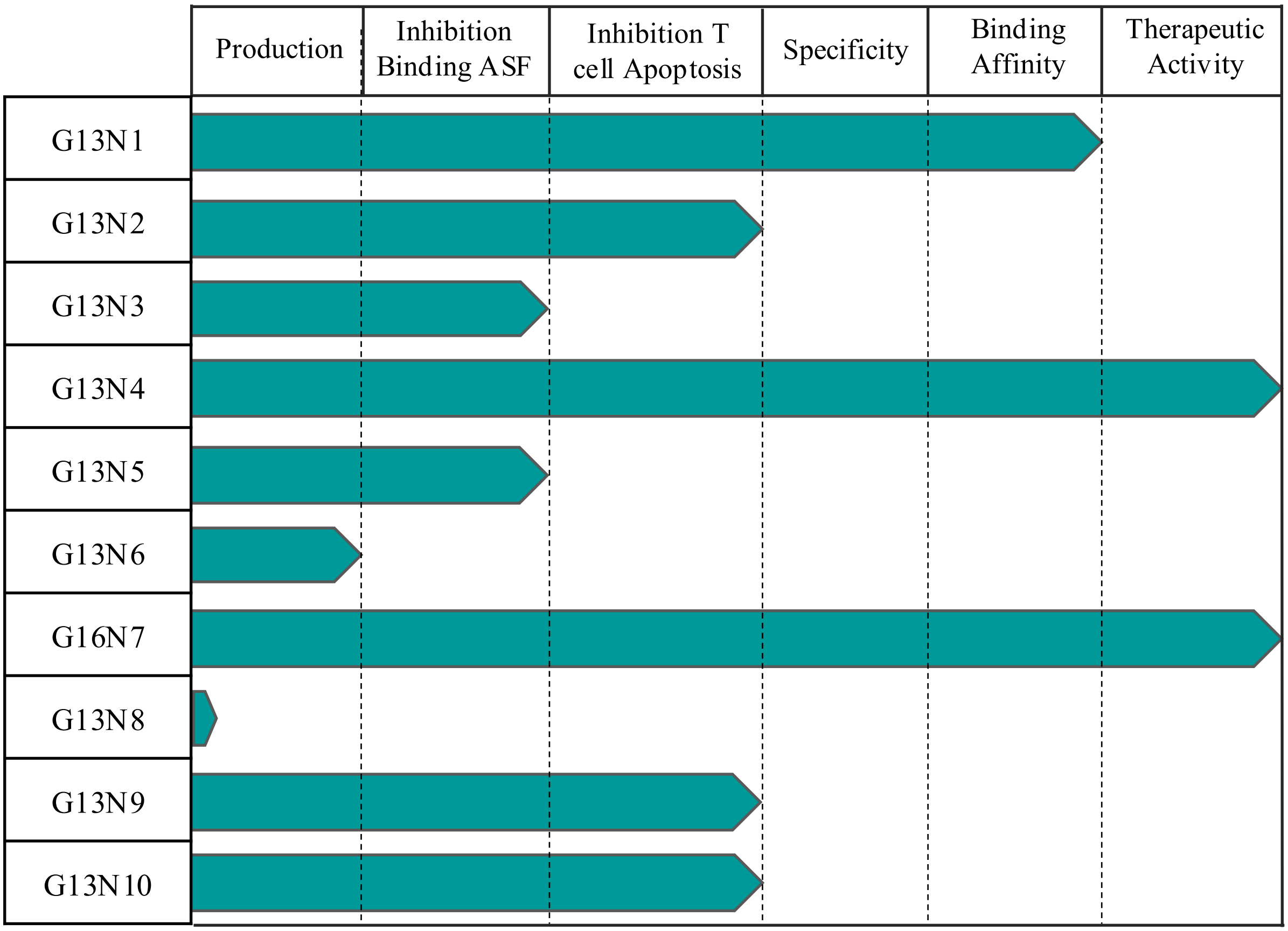

Figure S3. Hit-to-Lead process selecting Nbs targeting GAL-13.
